# Extracellular expression of glutamate decarboxylase B in *Escherichia coli* to improve gamma-aminobutyric acid production

**DOI:** 10.1186/s13568-016-0231-y

**Published:** 2016-08-22

**Authors:** Anqi Zhao, Xiaoqing Hu, Ye Li, Cheng Chen, Xiaoyuan Wang

**Affiliations:** 1 School of Biotechnology, Jiangnan University, Wuxi, 214122 China; 2 State Key Laboratory of Food Science and Technology, Jiangnan University, Wuxi, 214122 China; 3 Synergetic Innovation Center of Food Safety and Nutrition, Jiangnan University, Wuxi, 214122 China

**Keywords:** Gamma-aminobutyric acid, Glutamate decarboxylase, Secretory expression, TorA, GadB, *Escherichia coli*

## Abstract

*Escherichia coli* overexpressing glutamate decarboxylase GadB can produce gamma-aminobutyric acid with addition of monosodium glutamate. The yield and productivity of gamma-aminobutyric acid might be significantly improved if the overexpressed GadB in *E. coli* cells can be excreted outside, where it can directly transforms monosodium glutamate to gamma-aminobutyric acid. In this study, GadB was fused to signal peptides TorA or PelB, respectively, and overexpressed in *E. coli* BL21(DE3). It was found that TorA could facilitate GadB secretion much better than PelB. Conditions for GadB secretion and gamma-aminobutyric acid production were optimized in *E. coli* BL21(DE3)/pET20b-*torA*-*gadB*, leading the secretion of more than half of the overexpressed GadB. Fed-batch fermentation for GadB expression and gamma-aminobutyric acid production of BL21(DE3)/pET20b-*torA*-*gadB* was sequentially performed in one fermenter; 264.4 and 313.1 g/L gamma-aminobutyric acid were obtained with addition of monosodium glutamate after 36 and 72 h, respectively.

## Introduction

Gamma-aminobutyric acid (GABA) is the main inhibitory neurotransmitter in human cortex, it has been considered as a food constituent in Europe and a dietary supplement in USA (Boonstra et al. 2015; Dhakal et al. 2012; Wong et al. 2003). In addition to the usage in food additive and pharmaceuticals, GABA can also be used as the precursor for synthesizing the biodegradable polymer (Park et al. 2013).

GABA is usually synthesized from glutamic acid or glutamate in a reaction catalyzed by pyridoxal 5′-phosphate (PLP)-dependent enzyme glutamate decarboxylase (Gad, EC: 4.1.1.15). Direct synthesis of GABA from glucose in some microorganisms has been reported but the yield is usually low (Shi and Li 2011; Sun et al. 2008; Zhao et al. 2015). Addition of monosodium glutamate monohydrate (MSG) or glutamic acid in some microorganisms can efficiently improve GABA production. For example, *Lactobacillus brevis* NCL912 (Li et al. 2010), *Enterococcus avium* G-15 (Tamura et al. 2010) and *Lactobacillus buchneri* WPZ001 (Zhao et al. 2015) could produce 104, 116 and 129 g/L GABA from MSG, respectively.

Due to its importance, glutamate decarboxylase has been overexpressed in various bacteria to enhance GABA production. Since protein expression in *Escherichia coli* represents the most facile approach (Mahalik et al. 2014; Makino et al. 2011), overexpressing glutamate decarboxylase in *E. coli* became a good alternative for GABA production. For example, 5.07 and 5.69 g/L GABA could be produced from 10 g/L of MSG by overexpressing *Pyrococcus horikoshii* glutamate decarboxylase in *E. coli* XL1-Blue and a GABA aminotransferase mutant XL1-Blue ΔgabT, respectively (Le Vo et al. 2014). In *E. coli* K12 strains, there are two glutamate decarboxylases GadA and GadB encoded by *gadA* and *gadB,* respectively. Genes *gadA* and *gadB* are around 2200 kb apart on the chromosome, while *gadC,* encoding the glutamate/GABA antiporter GadC locates directly downstream of *gadB* (Ma et al. 2012, 2013). GadC imports glutamate into the cytoplasm, where GadA or GadB converts glutamate to GABA which is exported via GadC (Tsai et al. 2013). GABA can also be redirected into TCA cycle by GABA aminotransferase encoded by *gabT*. When GadA or GadB was overexpressed alone or together with GadC, GadB always showed higher activity. When GadB and GadC were co-overexpressed in *E. coli* XL1-Blue ΔgabT, 5.46 g/L GABA was obtained from 10 g/L MSG (Le Vo et al. 2012).

GABA production process in *E. coli* involves import of glutamate, formation and export of GABA, and GadB’s activity depends on PLP and pH (Yamada and O’Leary 1977), therefore, it is difficult to get high level GABA production yield. To solve this problem, recombinant *E. coli* overexpressing GadB was employed as a whole cell biocatalyst in buffer solution containing MSG. In this way, 94.8 g/L GABA was produced by concentrated XL1-Blue cells overexpressing GadB in 48 h (Park et al. 2013), and 280–300 g/L GABA was produced in 35 h by concentrated BL21(DE3) cells overexpressing GadA and treated with freezing and heating (Plokhov et al. 2000). Because GadA and GadB are intracellular enzymes, and most MSG are out of the cells, the recombinant *E. coli* cells have to be specially treated by sonication, ethyl acetate, toluene, and thermal activation to increase the permeability (Zhao et al. 2014). Therefore, if GadA and GadB can be secreted out of the cell, and efficiently contact with MSG, GABA production might be significantly improved.

The conserved secretory (Sec) pathway and twin-arginine translocation (Tat) pathway are two common protein secretion pathways in *E. coli* (Chen et al. 2014; Cheng et al. 2011; Choi and Lee 2004; Duan et al. 2013; Su et al. 2013). In Sec pathway, premature proteins containing signal peptide are exported to the periplasmic space where they are processed into mature proteins (Choi and Lee 2004; Dong et al. 2013); while in Tat system, proteins are directly secreted to the periplasmic space via a channel formed by Tat translocase (Berks 1996; Lee et al. 2006). Some recombinant proteins can be secreted by Tat pathway, but not by Sec system (Barrett et al. 2003; Dong et al. 2013). In this study, leader peptides TorA and PelB belonging to the Tat and Sec pathways, respectively, were chosen to be fused with GadB and overexpressed in *E. coli* BL21(DE3). We found that TorA could facilitate GadB secretion better than PelB. Under optimal conditions, more than half GadB in BL21(DE3)/pET20b-*torA*-*gadB* was secreted, and GABA could be efficiently synthesized form MSG added in the cultivation broth. 313.1 g/L GABA was produced when MSG was added in the fermentation broth of BL21(DE3)/pET20b-*torA*-*gadB* grown at 37 °C for 28 h and then heated to 50 °C at pH 4.6. Completion of both fermentation and bioconversion in the same fermentor simplifies the process for GABA production, which is convenient for industry application.

## Materials and methods

### Construction of *E. coli* strains BL21(DE3)/pET20b-gadB, BL21(DE3)/pET20b-torA-gadB, and BL21(DE3)/pET20b-pelB-gadB

**Fig. 1:**
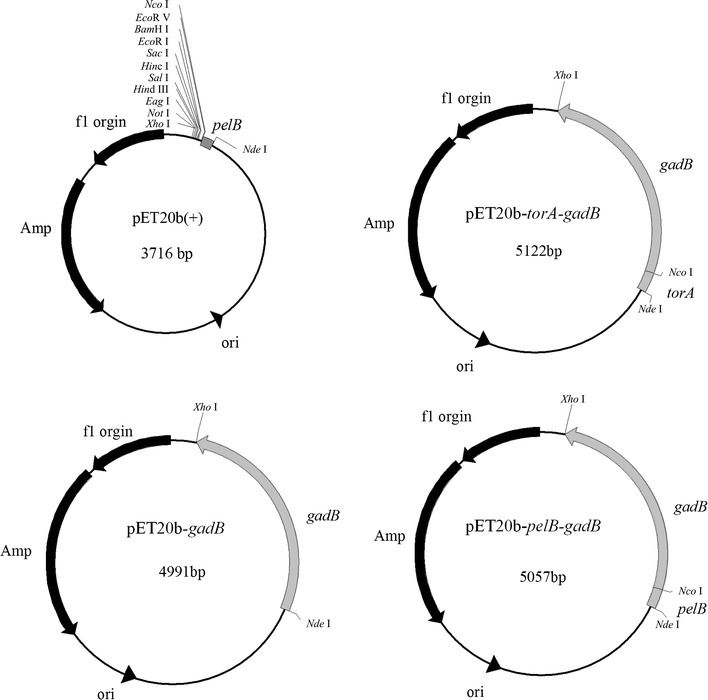
Maps of plasmids pET20b(+), pET20b-*gadB*, pET20b-*torA*-*gadB*, and pET20b-*pelB*-*gadB*

Strains and plasmids used in this study are listed in Table 1. Restriction enzymes, T4 DNA ligase and agarose gel DNA purification kit were purchased from TaKaRa (Dalian, China). Genomic DNA of *E. coli* W3110 was used as templates for PCR. Gene *gadB* (GenBank accession number: BAA15163.1) was amplified using primer pairs gadB-F1 (5′-CGCCATATGGATAAGAAGCAAGTAACG-3′) and gadB-R (5′-CCCTCGAGTCAGGTATGTTTAAAGCTGTT-3′), digested with *Nde*I and *Xho*I, and ligated into pET20b(+) that was similarly digested, resulting in pET20b-*gadB*, a plasmid harboring *gadB* without signal peptides. Gene *gadB* was amplified using primer pairs gadB-F2 (5′-CATCCATGGATAAGAAGCAAGTAACG-3′) and gadB-R, digested with *Nco*I and *Xho*I, and ligated into pET20b(+) that was similarly digested, resulting in pET20b-*pelB*-*gadB*, a plasmid harboring *gadB* with signal peptide *pelB*. Nucleotide *torA* encoding the signal peptide of trimethylamine-N-oxide reductase (GenBank accession number: BAA36139.1) was amplified using primer pairs torA-F (5′-CGCCATATGATGAACAATAACGATCTCTTTCAGGC-3′) and torA-R (5′-CATCCATGGCCGCTTGCGCCGCAGTC-3′), and digested with *Nde*I and *Nco*I. PCR products *torA* and *gadB* amplified using primers gadB-F1 and gadB-R were digested with *Nco*I and *Xho*I, and ligated into pET20b(+), resulting in pET20b-*torA*-*gadB*, a plasmid harboring *gadB* with signal peptide *torA* (Fig. 1). All plasmids were transformed into *E. coli* JM109 for selection and amplification, and into *E. coli* BL21(DE3) for protein expression.

**Table 1:** Strains and plasmids used in this study

Strains and plasmids	Description	Source
*Strains*		
W3110	Wild type *E. coli* strain ATCC 27325	ATCC
BL21(DE3)	*E. coli* strain for protein expression	Novagen
JM109	Host *E. coli* for gene cloning	Novagen
BL21(DE3)/pET20b(+)	BL21(DE3) containing pET20b(+)	This study
BL21(DE3)/pET20b-*gadB*	BL21(DE3) containing pET20b-*gadB*	This study
BL21(DE3)/pET20b-*pelB*-*gadB*	BL21(DE3) containing pET20b-*pelB*-*gadB*	This study
BL21(DE3)/pET20b-*torA*-*gadB*	BL21(DE3) containing pET20b-*torA*-*gadB*	This study
*Plasmids*		
pET20b(+)	*E. coli* protein expression vector	Novagen
pET20b-*gadB*	pET20b(+) harboring *gadB* without signal sequence	This study
pET20b-*pelB*-*gadB*	pET20b(+) harboring *gadB* with signal sequence *pelB*	This study
pET20b-*torA*-*gadB*	pET20b(+) harboring *gadB* with signal sequence *torA*	This study

### SDS-PAGE analysis and activity determination of GadB

A loop of *E. coli* cell colonies was inoculated into 20 mL LB medium (5 g/L yeast extract, 10 g/L tryptone and 10 g/L NaCl) in a 250-mL flask. 2 mL overnight seed culture was diluted into 50 mL TB medium (yeast extract 24 g/L, tryptone 12 g/L, glycerol 5 g/L, KH_2_PO_4_ 2.31 g/L, K_2_HPO_4_ 16.43 g/L) in a 250-mL flask and incubated for 36 h. When the optical density at 600 nm (OD_600_) reached 1.0, 1 mM isopropyl-β-d-1-thiogalactopyranoside (IPTG) was added to induce GadB expression. All the cultivations were conducted at 37 °C and 200 rpm, and 100 mg/L ampicillin was added for maintaining the plasmid. After induction for 36 h, 1 mL culture broth was centrifuged at 10,000×*g* for 10 min, the supernatant was collected as the extracellular fraction, and the cell pellets were washed and resuspended in 1 mL PBS buffer as the intracellular fraction.

For SDS-PAGE analysis, 20 μL protein samples were mixed with 5 μL 5× sample buffer (10 % SDS, 0.05 % bromophenol blue, 20 % glycerol, 10 mM beta-mercaptoethanol, and 0.2 M Tris–HCl, pH 6.8) and heated in boiling water for 10 min. Then 10 μL samples were loaded into the gel. Proteins in the gel were separated at 120 V for 1 h by electroporation using a Gene Pulser (Bio-Rad, USA) and stained with 0.25 % Coomassie brilliant blue R-250.

Activities of intracellular and extracellular GadB of BL21(DE3)/pET20b-*gadB*, BL21(DE3)/pET20b-*pelB*-*gadB* and BL21(DE3)/pET20b-*torA*-*gadB* were determined according to the published method (Shukuya and Schwert 1960) with minor modification. Cells were centrifuged. The supernatant was collected as the extracellular sample; the pellet was washed, resuspended in same volumes of PBS, sonicated to release the intracellular proteins, and used as the intracellular sample. The enzyme reaction mixture (1.0 mL) contains 0.2 M phosphate-citrate buffer (pH 4.6), 10 mM MSG and 0.1 mM PLP. The mixture was pre-incubated at 37 °C, and then 100 μL extracellular samples or 50 μL intracellular samples were added to start the reaction. The reaction mixture was incubated at 37 °C for 10 min, and then heated in boiling water for 10 min to quench the reaction. GABA and MSG concentrations in the mixture were measured using HPLC (Agilent Technologies 1200 series, USA) (Tamura et al. 2010) and Hypersil-ODS 5 μm (4.6 × 250 mm) column (Thermo, USA) according to the published method (Tamura et al. 2010). One unit of specific activity is defined as the amount of enzyme that produce 1 μmol of GABA in 1 min. When determining activities of samples from 3-L fermentor, 50 μL extracellular samples or 25 μL intracellular samples were added in the reaction mixture.

### Optimization of GadB secretory production in *E. coli* BL21(DE3)/pET20b-torA-gadB

Effects of cultivation temperatures (25, 30 and 37 °C), IPTG addition (0, 0.1, 0.4, 0.7, 1, 1.5 or 2 mM), glycine addition (0, 2.5, 5, 7.5, 10, 12.5, 20 or 30 g/L) and NaCl addition (0, 2.5, 5, 10 or 15 g/L) on GadB secretory expression of BL21(DE3)/pET20b-*torA*-*gadB* were investigated.

### Effects of temperature on activity and stability of GadB in the broth

Since the cell cultivation broth was directly used for GABA production, effect of temperature on activity and stability of the crude GadB in the broth was analyzed.

A loop of cell colonies of BL21(DE3)/pET20b-*torA*-*gadB* was inoculated into 20 mL LB medium in a 250-mL flask and grown overnight. 2 mL overnight culture was diluted into 50 mL TB medium supplied with 7.5 g/L glycine and 5 g/L NaCl in a 250-mL flask. When OD_600_ reached 1.0, 0.7 mM IPTG was added. Cells were incubated at 37 °C and 200 rpm; 100 mg/L ampicillin was added to maintain the plasmid.

After 36 h, 100 μL broth culture was taken and added into the pre-incubated enzyme reaction mixture at 22, 30, 37, 45 or 50 °C, and incubated at the same temperature for 10 min; then GadB activity was determined at these temperature.

Stability of GadB in the broth condition was determined at 37 or 50 °C. After 36 h fermentation, GadB was produced in the broth. Then pH of the culture broth was adjusted to 4.6 by H_3_PO_4_. pH 4.6 is the optimum pH for GadB (Biase et al. 1996; Jun et al. 2014; Shukuya and Schwert 1960) but *E. coli* growth could be inhibited at pH 4.6. Temperature was kept to 37 or 50 °C for another 36 h. 100 μL sample was taken at different time points to determine the residual GadB activity. Stability of GadB was evaluated by the ratio (relative activity) of the residual GadB activity at different time points to the activity at the beginning.

### GadB expression and GABA synthesis at flask-shaking scale

Overnight culture of BL21(DE3)/pET20b-*torA*-*gadB* was diluted into 50 mL TB medium supplied with 7.5 g/L glycine and 5 g/L NaCl in a 250-mL flask. When OD_600_ reached 1.0, 0.7 mM IPTG was added. Cells were incubated at 37 °C and 200 rpm; 100 mg/L ampicillin was added to maintain the plasmid.

After 36 h expression and secretion of GadB, two portions of 50 mL cell culture of BL21(DE3)/pET20b-*gadB* or BL21(DE3)/pET20b-*torA*-*gadB* were collected. One portion was used as the whole sample; while the other portion was centrifuged, the supernatant was collected as the extracellular sample, and the cell pellets was resuspended in the same volumes of PBS buffer and used as the intracellular sample. GABA synthesis was initiated by adding 0.1 mM PLP and 25 g MSG in the broth. MSG was over-saturated at the beginning, but gradually dissolved with its conversion to GABA. The temperature was set at either 37 or 50 °C. pH was adjusted manually to 4.6, the optimal pH for GadB activity (Biase et al. 1996; Shukuya and Schwert 1960), by adding 50 % H_3_PO_4_ every 30 min at the first 6 h and every 2 h afterwards. GABA productions in the three samples were determined at different time points.

### Combination of GadB expression and GABA production in the same bioreactor

100 mL seed culture in TB medium supplemented with 5 g/L NaCl, 7.5 g/L glycine and 100 mg/L ampicillin was prepared in 500-mL flasks at 37 °C and 200 rpm for 8 h, and then transferred to a 3-L fermentor (New Brunswick Scientific BioFlo 110, USA) containing 1.0 L fermentation medium (Duan et al. 2013; Neubauer et al. 2007). The fermentation medium contains 30.0 g/L tryptone, 20.0 g/L yeast extract, 2.0 g/L Na_2_SO_4_, 2.5 g/L (NH_4_)_2_SO_4_, 1.0 g/L diammonium hydrogen citrate, 14.6 g/L K_2_HPO_4_, 3.6 g/L NaH_2_PO_4_·2H_2_O, 2.0 g/L MgSO_4_·7H_2_O, 100 mg/L thiamine, 8.0 g/L glycerol, and 1.0 mL/L trace metal solution, pH 7.0. The feeding nutrition solutions included 500 g/L glycerol, 3.4 g/L MgSO_4_·7H_2_O, 50 g/L yeast extract, and 50 g/L tryptone.

Since acetate, an extracellular byproduct of aerobic fermentation, inhibits cell growth and recombinant protein formation (Shiloach and Fass 2005), glycerol was fed with an increasingly exponential feeding rate before IPTG induction (Cheng et al. 2011; Fang et al. 2011) and the total amount of glycerol used during the fermentation was about 180 g. When OD_600_ reached 15, 7.5 g/L glycine and 5.0 g/L NaCl was supplied (Cheng et al. 2011). When OD_600_ reached 50, 0.7 mM IPTG was added and glycerol feeding rate switched to 12 mL/h. The air flow rate was set at 1.8 L/min, temperature was set at 37 °C, pH was kept at 7.0 by automatic addition of ammonia solution (25 %, v/v) and antifoam was added manually when necessary. The DO level was maintained around 30 % of air saturation by varying the agitation speed from 300 to 900 rpm. Residual glycerol in the broth was measured using HPLC (Agilent Technologies 1200 series, USA) according to the published method (Tamura et al. 2010). The space-time-yield (SPY) is defined as the ratio of GABA production to the total time used for GadB expression and GABA production. Conversion rate of MSG to GABA is defined as the mole ratio of the produced GABA to the added MSG.

After 28 h, pH was adjusted to 4.6 by 50 % H_3_PO_4_, agitation speed was set to 200 rpm, and temperature was kept at 50 or 37 °C. PLP were added to the final concentration of 1 mM, and 650 g dry MSG was added in several batches.

## Results

### Signal peptide TorA facilitates GadB secretions in *E. coli*

**Fig. 2:**
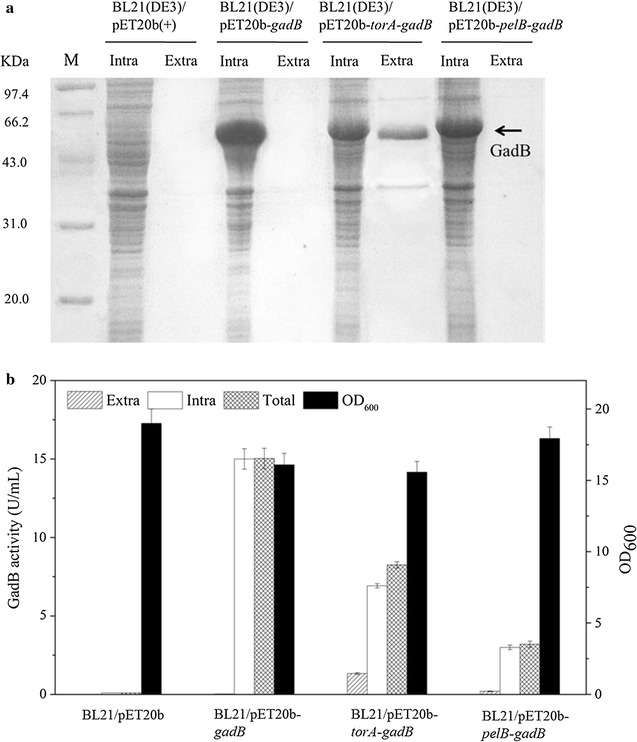
BL21(DE3)/pET20b(+), BL21(DE3)/pET20b-*gadB*, BL21(DE3)/pET20b- *torA*-*gadB* and BL21(DE3)/pET20b-*pelB*-*gadB* cells were grown in TB medium at 37 °C for 36 h. **a** The intracellular and extracellular fractions from different cells were analyzed by using SDS-PAGE. **b** Intracellular and extracellular gadB activities and cell densities of different strains were determined. The molecular mass of GadB is about 53 kDa. *M* marker of standard protein; *Intra* intracellular sample; *Extra* extracellular sample; *Total* the sum GadB activity of intracellular and extracellular samples

GABA production involves import of glutamate, formation and export of GABA, therefore, it would be improved if GadB was secreted out of the cells. In this study, leader peptides TorA and PelB belonging to the Tat and Sec pathways, respectively, were chosen to be fused with GadB. *E. coli* strains BL21(DE3)/pET20b(+), BL21(DE3)/pET20b-*gadB*, BL21(DE3)/pET20b-*torA*-*gadB*, and BL21(DE3)/pET20b-*pelB*-*gadB* (Fig. 1) were grown, and intracellular and extracellular protein samples were prepared and analyzed by SDS-PAGE (Fig. 2a). A strong protein band around 53 kDa, the size expected for GadB, was observed in the intracellular protein samples from BL21(DE3)/pET20b-*gadB*, BL21(DE3)/pET20b-*torA*-*gadB* and BL21(DE3)/pET20b-*pelB*-*gadB*, but not in that from BL21(DE3)/pET20b(+), suggesting GadB were overexpressed in these cells. GadB protein band was also observed in the extracellular protein samples from BL21(DE3)/pET20b-*torA*-*gadB,* but not in other extracellular protein samples, suggesting that TorA facilitates GadB secretion better than PelB.

GadB activities of the intracellular and extracellular samples from *E. coli* strains BL21(DE3)/pET20b(+), BL21(DE3)/pET20b-*gadB*, BL21(DE3)/pET20b-*torA*-*gadB*, and BL21(DE3)/pET20b-*pelB*-*gadB* were determined, respectively (Fig. 2b). Neither intracellular nor extracellular activity of GadB was observed for samples from BL21(DE3)/pET20b(+), consistent with the fact that the expression of chromosomal *gadB* in *E. coli* is controlled by acid respond system (Feehily and Karatzas 2013). When GadB was overexpressed using plasmid in BL21(DE3)/pET20b-*gadB,* the intracellular activity of GadB reached 15.0 U/mL, but no extracellular activity was observed, indicating that GadB cannot be secreted by itself in *E. coli*. When GadB was fused to TorA in BL21(DE3)/pET20b-*torA*-*gadB*, 6.9 U/mL intracellular activity and 1.3 U/mL extracellular activity were observed. However, when GadB was fused to the peptide PelB in BL21(DE3)/pET20b-*pelB*-*gadB*, 3.2 U/mL intracellular activity and trace extracellular activity, 0.14 U/mL, was observed. This suggests again that TorA facilitates GadB secretion much better than PelB in *E. coli*. Even though similar GadB proteins were expressed in BL21(DE3)/pET20b-*torA*-*gadB* and BL21(DE3)/pET20b-*pelB*-*gadB,* total GadB activity in the former was much higher than in the latter. This indicates that some GadB proteins overexpressed in BL21(DE3)/pET20b-*pelB*-*gadB* might not be functional. PelB is a signal peptide of Sec pathway. In Sec pathway, premature proteins containing signal peptide are exported to the periplasmic space where they are processed into mature proteins (Choi and Lee 2004; Dong et al. 2013). GadB was not secreted extracellular in Sec pathway using pelB signal sequence, possibly because it contains 6 subunits and such a big protein is not easier to be correctly folded in the limited periplasmic space. TorA, a signal peptide of Tat system, can efficiently secreted GadB in *E. coli* BL21(DE3) (Berks 1996; Lee et al. 2006). Therefore, BL21(DE3)/pET20b-*torA*-*gadB* was used for further investigation.

### Optimization of GadB secretory production in *E. coli* BL21(DE3)/pET20b-torA-gadB

Major factors affecting GABA production by microbial fermentation are temperature, pH, fermentation time and additives (Fang et al. 2011). In BL21(DE3)/pET20b-*torA*-*gadB,* only a portion of GadB proteins was secreted and their activities were low, therefore, the cultivation conditions were optimized.

**Fig. 3:**
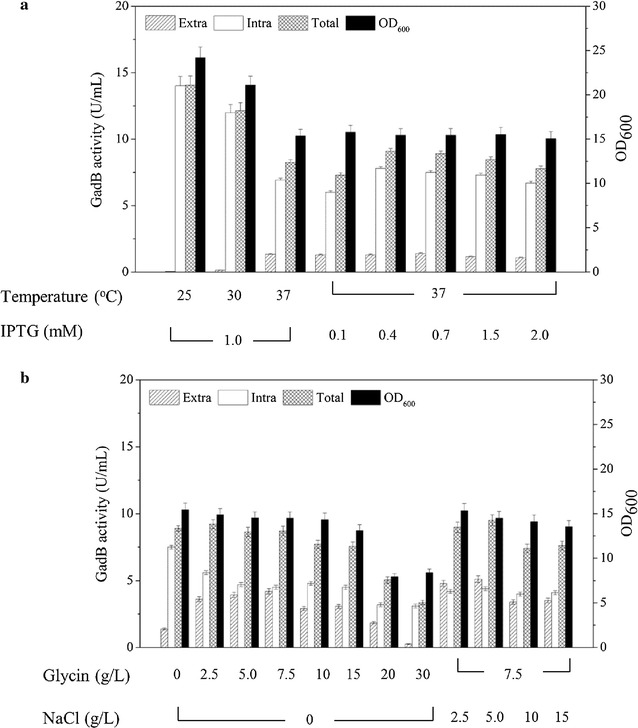
Optimization of GadB expression and secretion in BL21(DE3)/pET20b-*torA*-*gadB*. **a** Effect of temperature and IPTG on cell density, intracellular and extracellular activities. **b** Effect of NaCl and Glycine on cell density, intracellular and extracellular activities. Cells were grown in TB medium at 37 °C for 36 h. The *error bars* indicate the standard deviations from three parallel experiments

BL21(DE3)/pET20b-*torA*-*gadB* was cultured at 25, 30 and 37 °C, respectively, and GadB activities of the intracellular and extracellular samples were determined. As shown in Fig. 3a, OD_600_ and total GadB activity decreased with increase of temperature, but the highest extracellular GadB activity (1.33 U/mL) was observed for cells grown at 37 °C. This is quite different from the protein secretion through the Sec system which decreases with increase of temperature (Duan et al. 2013; Li et al. 2014). While the extracellular activities of GadB from BL21(DE3)/pET20b-*pelB*-*gadB* cells grown at 25 and 30 °C were 1.12 and 0.77 U/mL, respectively. In the Sec system, immature polypeptide chains are exported to the periplasmic space where they are folded into mature type. At low temperature, the synthetic rate of immature proteins decreases, which provides enough time for them to translocate and fold correctly without forming inclusion bodies, thus secretory proteins increase via the Sec pathway (Jong et al. 2010; Li et al. 2014). In BL21(DE3)/pET20b-*torA*-*gadB,* GadB was secreted via the Tat system in which the correctly folded proteins are directly translocated, and at higher, relevant enzymes are more active and membrane fluidity is better, so the highest extracellular GadB activity was observed for BL21(DE3)/pET20b-*torA*-*gadB* cells grown at 37 °C. Without the protection of cell membrane, the stability of extracellular GadB decreases, therefore, the total GadB activity for BL21(DE3)/pET20b-*torA*-*gadB* cells grown at 37 °C was lower than that of the cells grown at 25 or 30 °C (Fig. 3a).

Effect of IPTG concentration (ranging from 0.1 to 2.0 mM) on the expression of the *torA*-*gadB* at 37 °C was investigated (Fig. 3a). IPTG concentration only slightly affects the cell growth and GadB activity in BL21(DE3)/pET20b-*torA*-*gadB*. The highest extracellular GadB activity (1.39 U/mL) was found in BL21(DE3)/pET20b-*torA*-*gadB* with 0.7 mM IPTG induction.

More periplasmic proteins in *E. coli* could leach into medium by disrupting the outer membrane integrity, using gene modification (Wan and Baneyx 1998) or osmolyte supplementation (de Marco et al. 2005). In this study, glycine supplementation increases membrane permeability of *E. coli* by disrupting the synthesis of peptidoglycan cross-linkages (Choi and Lee 2004). Therefore, effect of glycine on cell growth and GadB activity in BL21(DE3)/pET20b-*torA*-*gadB* was investigated (Fig. 3b). When up to 7.5 g/L glycine was added, both OD_600_ and total GadB activities only slightly changed, but the extracellular GadB activity significantly increased. When more than 7.5 g/L glycine was added, values of OD_600_, total activity and extracellular activity of GadB in BL21(DE3)/pET20b-*torA*-*gadB* decreased. When 30 g/L glycine was added, OD_600_ only reached 7.94, and extracellular GadB activity was only 0.26 U/mL, suggesting that too much glycine inhibits the cell growth and GadB secretion in BL21(DE3)/pET20b-*torA*-*gadB.* The highest extracellular GadB activity (4.20 U/mL) was found in BL21(DE3)/pET20b-*torA*-*gadB* with 7.5 g/L glycine addition.

NaCl could increase the conversion rate of GadB from the neutral-pH inactive form into the low-pH active form (Capitani et al. 2003; Gut et al. 2006; O’Leary and Brummund 1974), and enhance GadB stability (Jun et al. 2014). Thus, the influence of NaCl (2.5, 5.0, 1.0 and 15 g/L) on cell growth and GadB activity in BL21(DE3)/pET20b-*torA*-*gadB* was also investigated in the media containing 7.5 g/L glycine (Fig. 3b). The highest extracellular GadB activity (5.11 U/mL) was obtained in BL21(DE3)/pET20b-*torA*-*gadB* with addition of 5.0 g/L NaCl.

### Effects of temperature on the activities and stability of GadB in the broth

Active GadB proteins overexpressed in BL21(DE3)/pET20b-*torA*-*gadB* were secreted, and MSG could be directly added to the broth to produce GABA. This would simplify the process for GABA production and have potential industry application. Therefore, it is necessary to test the activity and stability of the secreted GadB in broth.

**Fig. 4:**
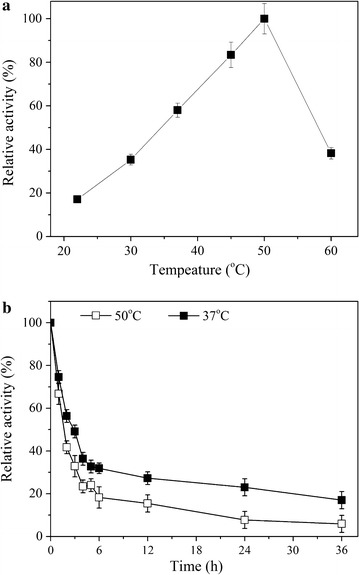
Temperature and stability of the GadB of BL21(DE3)/pET20b-*torA*-*gadB* in the broth. **a** Effect of temperature on GadB activity. **b** Stability of GadB in TB broth with 7.5 g/L glycine and 5.0 g/L NaCl. The *error bars* indicate the standard deviations from three parallel experiments

BL21(DE3)/pET20b-*torA*-*gadB* cells were grown for 36 h under optimal conditions (Biase et al. 1996; Jun et al. 2014; Shukuya and Schwert 1960), and then the broth sample was taken for determining the GadB activity at 22, 30, 37, 45, 50 or 60 °C (Fig. 4a). At temperatures below 50 °C, GadB activity increases with temperature, but it dropped at 60 °C. The highest GadB activity was obtained when the reaction was performed at 50 °C, which is 60 % higher than the activity at 37 °C.

To investigate the stability of the active GadB in broth at 50 °C, the sample was incubated at 50 °C for 36 h and GadB activity was measured at different time points, using the sample incubated at 37 °C as control. As shown in Fig. 4b, GadB activity decreased with time and at all time points, residual GadB activity in the sample incubated at 50 °C was less than the sample incubated at 37 °C. After 6 h incubation, 29.5 and 16.9 % residual GadB activity were remained for samples incubated at 37 and 50 °C, respectively; after 36 h incubation, only 17.3 and 5.9 % residual GadB activity were remained for samples incubated at 37 and 50 °C, respectively. This indicated that extracellular GadB in BL21(DE3)/pET20b-*torA*-*gadB* was more stable at 37 °C than at 50 °C.

### Combination of GadB expression and GABA synthesis at shaking flask scale

**Fig. 5:**
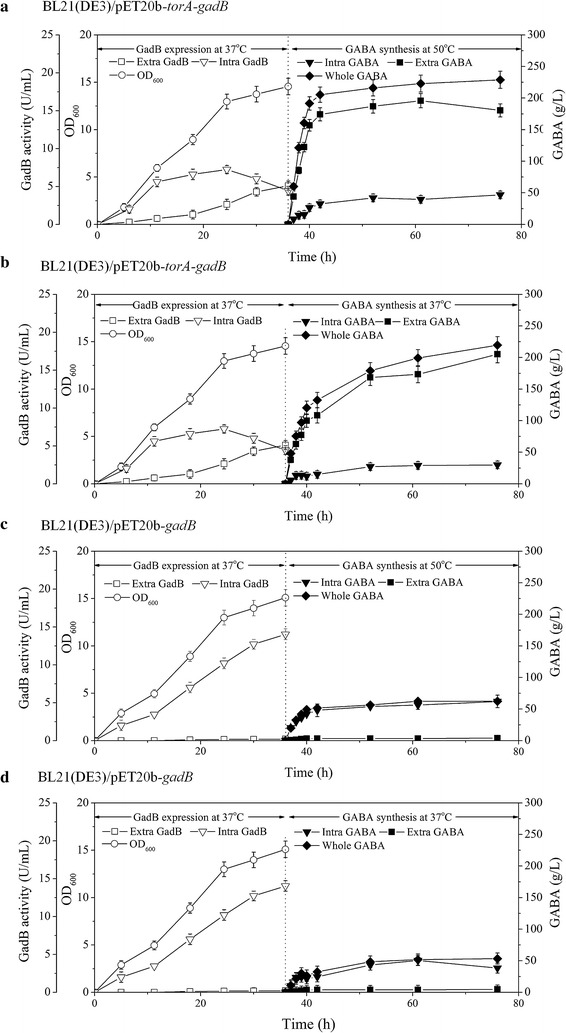
Comparison of GadB secretion at 37 °C and GABA production at 50 and 37 °C for BL21(DE3)/pET20b-*torA*-*gadB* cells, using BL21(DE3)/pET20b-*gadB* cells as control. Cells were grown at 37 °C for 36 h for expression and secretion of GadB. Then two portions of 50 mL cell culture were collected. One portion was used as the whole sample; while the other portion was centrifuged, the supernatant was collected as the extracellular sample, and the cell pellets was resuspended in the same volumes of PBS buffer and used as the intracellular sample. At this time point (the 36 h time point), GABA synthesis was initiated by adding 25 g MSG in the broth. pH was adjusted manually to 4.6. GABA productions were determined at different time points. **a** BL21(DE3)/pET20b-*torA*-*gadB* grown at 37 °C produced GABA at 50 °C. **b** BL21(DE3)/pET20b-*torA*-*gadB* grown at 37 °C produced GABA at 37 °C. **c** BL21(DE3)/pET20b-*gadB* grown at 37 °C produced GABA at 50 °C. **d** BL21(DE3)/pET20b-*gadB* grown at 37 °C produced GABA at 37 °C

BL21(DE3)/pET20b-*gadB* and BL21(DE3)/pET20b-*torA*-*gadB* were grown in flask under the optimal conditions for 36 h; cell density, extracellular and intracellular GadB activities were determined. Similar growth curves were observed for both strains; cell density kept increasing until 36 h. Extracellular and intracellular GadB activities for both strains were quite different. Then two portions of 50 mL cell culture were collected. One portion was used as the whole sample; while the other portion was centrifuged, the supernatant was collected as the extracellular sample, and the cell pellets was resuspended in the same volumes of PBS buffer and used as the intracellular sample. GABA synthesis was initiated by adding 0.1 mM PLP and 25 g MSG in the broth. MSG was over-saturated at the beginning, but gradually dissolved with its conversion to GABA (Fig. 5). The temperature was set at either 37 or 50 °C. pH was adjusted manually to 4.6, the optimal pH for GadB activity, by adding 50 % H_3_PO_4_ every 30 min at the first 6 h and every 2 h afterwards. GABA productions in the three samples were determined at different time points.

For BL21(DE3)/pET20b-*torA*-*gadB,* intracellular GadB activity quickly increased at the first 12 h, then slowed down, reaching the peak (7.25 U/mL) at 24 h, and gradually decreased afterwards; extracellular GadB activity continuously increased and exceeded the intracellular GadB activity around 36 h, reaching the maximum (5.11 U/mL) at 36 h. For BL21(DE3)/pET20b-*gadB,* intracellular GadB activity continuously increased, reaching 14.04 U/mL at 36 h, but little extracellular GadB activity was observed.

At 50 °C, GABA in the whole or the extracellular samples of BL21(DE3)/pET20b-*torA*-*gadB* was promptly accumulated in from 36 to 42 h, reaching 205.3 and 174.1 g/L at 42 h, respectively. The conversion rate of MSG to GABA for the whole or the extracellular samples of BL21(DE3)/pET20b-*torA*-*gadB* at 42 h reached 0.74 and 0.63 mol/mol, respectively. GABA production of the whole sample reached 228.9 g/L at 76 h, and the MSG to GABA conversion rate reached 0.83 mol/mol. But for the intracellular sample, only 46.8 g/LGABA in was accumulated after 76 h and the MSG to GABA conversion rate only reached 0.17 mol/mol (Fig. 5a).

At 37 °C, GABA production of BL21(DE3)/pET20b-*torA*-*gadB* was slower but enduring. GABA production in the whole and the extracellular samples were 132.6 and 108.2 g/L at 42 h, respectively, and the MSG to GABA conversion rate reached 0.48 and 0.39 mol/mol, respectively. GABA production of the whole sample reached 219.6 g/L after 76 h, and the MSG to GABA conversion rate reached 0.80 mol/mol. For the intracellular sample, only 29.9 g/L GABA in was accumulated, and the MSG to GABA conversion rate only reached 0.11 mol/mol after 76 h (Fig. 5b).

For BL21(DE3)/pET20b–*gadB*, GABA production in the extracellular sample was low, only 4.7 and 3.8 g/L GABA were produced after 76 h at 50 and 37 °C, respectively (Fig. 5c, d). GABA production of the intracellular samples of BL21(DE3)/pET20b–*gadB* was higher than that of BL21/pET20b-*torA*-*gadB*, but GABA production of the whole samples of BL21(DE3)/pET20b-*gadB* was much lower than that of BL21(DE3)/pET20b-*torA*-*gadB*. The results demonstrate that the extracellular expression of GadB plays major role in the high GABA production of BL21(DE3)/pET20b-*torA*-*gadB.*

### Combination of GadB expression and GABA synthesis in bioreactor

**Fig. 6:**
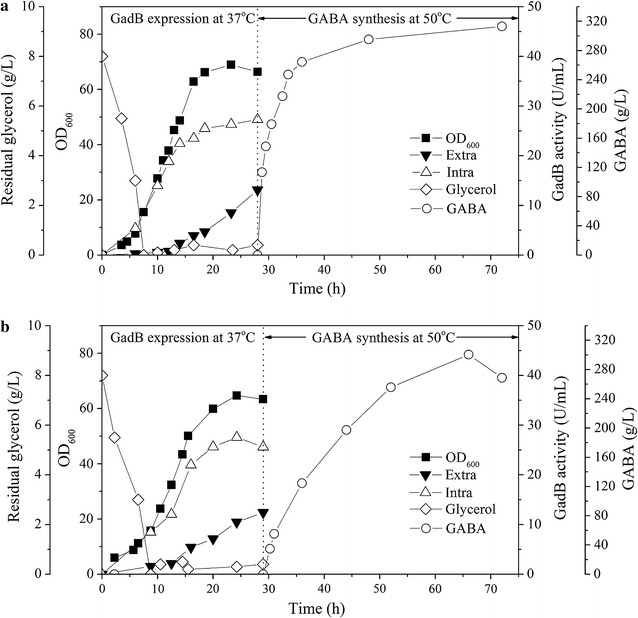
Fed-batch fermentation for GadB expression and GABA production of BL21(DE3)/pET20b-*torA*-*gadB*. GadB expression and GABA fermentation was performed in the same fermenter. The fermentation was done in two terms; (1) Culture for expression of GadB (without producing GABA). (2) Production of GABA with addition of MSG to the medium (with or without heat activation). Cells were grown at 37 °C for 28 h for expression and secretion of GadB. Then pH was adjusted to 4.6 and temperature was kept at 50 or 37 °C, and 650 g dry MSG was added in several batches in the same bioreactor. **a** Time profile of GadB expression at 37 °C and GABA production at 37 °C. **b** Time profile of GadB expression at 37 °C and GABA production at 50 °C. *Curves* of residual glycerol, cell density, intracellular and extracellular GadB activities, and GABA concentrations are shown

In order to further enhance GABA production, fed-batch fermentations of BL21(DE3)/pET20b-*torA*-*gadB* was conducted. BL21(DE3)/pET20b-*torA*-*gadB* were grown in a 3-L fermenter; cell density, extracellular and intracellular GadB activities were determined. Then GABA production was performed in the same fermenter (Fig. 6). The intracellular GadB activity increased promptly, reaching 22.45 U/mL at 14 h, and then slightly increased to 27.29 U/mL at 28 h. The extracellular GadB activity quickly increased after IPTG induction at 14 h and reached 13.12 U/mL at 28 h. At 28 h, 0.1 mM PLP and 650 g MSG were added, pH was adjusted to 4.6, and the temperature was set at 50 °C (Fig. 6a). GABA was promptly accumulated from 28 to 36 h, reached 264.4 g/L at 36 h, and then slowly increased to 313.1 g/L at 72 h. The space-time-yield (SPY) quickly increased during 28–36 h, reaching 7.37 g/L/h at 34 h and 7.34 g/L/h at 36 h, respectively. Conversion rate of MSG to GABA reached 0.80 mol/mol at 36 h and 0.95 mol/mol at 72 h, respectively.

GABA production at 37 °C was also investigated (Fig. 6b). GABA production reached 124.5 g/L at 36 h and 300.4 g/L at 66 h. The maximum SPY is 4.91 at 52 h. Conversion rate of MSG to GABA reached 0.37 mol/mol at 36 h and 0.91 mol/mol at 72 h, respectively.

## Discussion

*E. coli* is one of the most widely used hosts for the secretory production of secretory recombinant proteins and these proteins also has several advantages, such as simplicity of purification and better stability (Choi and Lee 2004). GABA can be produced from MSG by *E. coli* overexpressing GadB, but the substrate has to enter the cell and the product has to be exported out of the cell. In this study, we found that TorA could facilitate GadB secretion in *E. coli* BL21(DE3)/pET20b-*torA*-*gadB*. Under optimal conditions, more than half GadB was secreted. After the fermentation combining GadB expression and GABA production, 264.4 and 313.1 g/L GABA was produced at 36 h and 72 h, respectively. As we know, this is the highest GABA production yield in *E. coli*.

Previous reports on GABA production in *E. coli* were performed by intracellular overexpressing Gad and other relevant enzymes or improving methods of fermentation. To increase GABA production, whole cell biocatalyst method was adopted. Recombinant *E. coli* cells were treated by freezing at −20 °C and heating at 53 °C before used for bioconversion. The highest GABA production could reach 280–300 g/L in 35 h (Plokhov et al. 2000). In this study, the extracellular expression of GadB would simplify the process of enzyme purification and immobilization for GABA production, and the fermentation broth can also be directly used for GABA production without cell separation and GadB purification. Most importantly, in this study, both GadB expression and GABA production were completed in the same fermenter, which is convenient for industry application.

GABA can be produced by either *E. coli* or lactic acid bacteria. *E. coli* cells contain endotoxin, therefore GABA has to be separated from the *E. coli* cells when used as food additives; while lactic acid bacteria is safe and can be used without separation with GABA. It seems that lactic acid bacteria are more appropriate for the production of GABA. But production of GABA in *E. coli* can be much higher than that in lactic acid bacteria. In this study, 264 g/L GABA can be obtained in 36 h by *E. coli* fermentation, while the highest GABA production in lactic acid bacteria reported is 129 g/L with 60 h fermentation and bioconversion (Zhao et al. 2015). Endotoxin contaminated from *E. coli* cells can also be removed by downstream process (Lopes et al. 2010). Because of its high yield in *E. coli*, the production cost of GABA using *E. coli* might be less than that using lactic acid bacteria. When used as the precursor for some biopolymer, GABA produced by *E. coli* has more potential.

## Abbreviations

GABA: gamma-aminobutyric acid
GadB: glutamate decarboxylase B
Gad: glutamate decarboxylase
MSG: monosodium glutamate monohydrate
IPTG: isopropyl-β-d-1-thiogalactopyranoside
SPY: space-time-yield
PLP: pyridoxal 5′-phosphate
Tat pathway: twin-arginine translocation pathway
Sec pathway: secretory pathway
TorA: signal peptide of trimethylamine-N-oxide reductase
